# 

**Published:** 1879-05-20

**Authors:** 


☞ TO EVERY SUBSCRIBER who has not paid his subscription we respectfully and kindly say we are needing money. Please remit. R. C. WORD.

